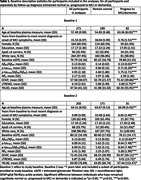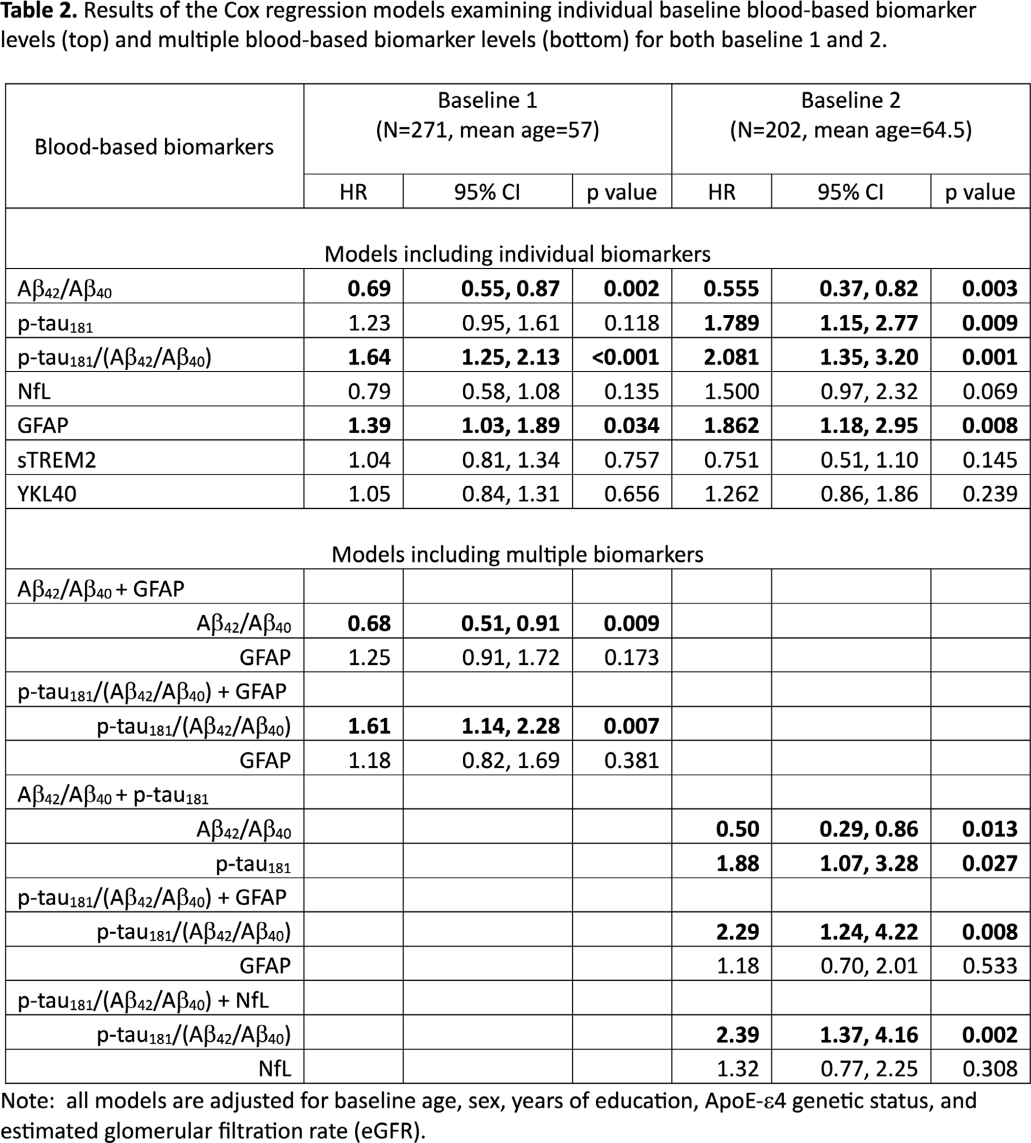# Blood‐based biomarkers and risk of MCI symptom onset over the short and long‐term

**DOI:** 10.1002/alz.091476

**Published:** 2025-01-09

**Authors:** Anja Soldan, Corinne Pettigrew, Jiangxia Wang, Marilyn S. Albert, Kaj Blennow, Tobias Bittner, Abhay Moghekar

**Affiliations:** ^1^ Johns Hopkins University School of Medicine, Baltimore, MD USA; ^2^ Johns Hopkins Bloomberg School of Public Health, Baltimore, MD USA; ^3^ University of Gothenburg, Gothenburg Sweden; ^4^ Roche Pharmaceuticals, Basel Switzerland

## Abstract

**Background:**

Blood‐based biomarkers of amyloid and tau have been shown to predict AD‐dementia risk. Much less is known about their ability to predict risk of Mild Cognitive Impairment (MCI) among cognitively normal individuals. It is also unclear how AD non‐specific blood markers of neurodegeneration and neuroinflammation predict MCI and whether they add predictive power above and beyond AD biomarkers. The current study examined whether levels of blood biomarkers of amyloid (Ab_42_/Ab_40_), tau (p‐tau_181_), neurodegeneration (NfL), and neuroinflammation (GFAP, YKL40, sTREM2) collected when participants were cognitively normal predict the time to onset of MCI, both alone and in combination.

**Method:**

The plasma assays were based on the NeuroToolKit (cobas Elecsys assays, Roche Diagnostics) and obtained from 271 cognitively unimpaired BIOCARD Study participants at their initial baseline evaluation (mean age=57.5y, including 82 who progressed to MCI/dementia). A second ‘baseline’ specimen (collected using different procedures) was evaluated for a subset of participants who were cognitively normal ∼7 years later (N=202) (mean age = 64.5y), including 31 who later developed MCI/dementia, Table 1). Mean clinical follow‐up was 15.5 years for Baseline 1 and 9.9 years for ‘Baseline 2’. Cox regression models tested the association of biomarker levels with time to MCI symptom onset, separately for both baselines.

**Results:**

For both baselines, lower levels of Ab_42_/Ab_40_, higher GFAP, and a higher ratio of p‐tau_181_/Ab_42_/Ab_40_were each associated with an earlier time to MCI symptom onset (p’s<=0.034, Table 2). For baseline 2, higher p‐tau_181_ (p=0.009) was also associated with earlier MCI symptom onset (p=0.009), and higher NfL was associated with earlier MCI symptom onset for progression within 7 years (p=0.05). When combining biomarkers, Ab_42_/Ab_40_ and p‐tau_181_ independently predicted progression (p’s <0.03), but neither GFAP nor NFL added predictive value after accounting for Ab_42_/Ab_40_ and p‐tau_181_. YKL40 and sTREM2 were not associated with MCI onset in any analyses.

**Conclusion:**

This indicate that during preclinical AD, more abnormal blood biomarker levels of amyloid (Ab_42_/Ab_40_), tau (p‐tau_181_), neurodegeneration (NfL), and neuroinflammation (GFAP) individually predict progression from normal cognition to MCI, but that AD non‐specific neurodegeneration and inflammation do not improve prediction after accounting for amyloid and p‐tau levels.